# White Paper for Global Palliative Care Advocacy: Recommendations from a PAL-LIFE Expert Advisory Group of the Pontifical Academy for Life, Vatican City

**DOI:** 10.1089/jpm.2018.0248

**Published:** 2018-10-09

**Authors:** Carlos Centeno, Thomas Sitte, Liliana de Lima, Sami Alsirafy, Eduardo Bruera, Mary Callaway, Kathleen Foley, Emmanuel Luyirika, Daniela Mosoiu, Katherine Pettus, Christina Puchalski, M.R. Rajagopal, Julianna Yong, Eduardo Garralda, John Y. Rhee, Nunziata Comoretto

**Affiliations:** ^1^ PAL-LIFE Project—Pontifical Academy for Life, International Advisory Group for Global Palliative Care Advocacy, Vatican City.; ^2^ ATLANTES Research Programme, Institute for Culture and Society, University of Navarra, Pamplona, Spain.; ^3^ Deutsche PalliativStiftung, Fulda, Germany.; ^4^ International Association for Hospice and Palliative Care (IAHPC), Houston, Texas.; ^5^ Palliative Medicine Unit, Kasr Al-Ainy School of Medicine, Cairo University, Cairo, Egypt.; ^6^ Department of Palliative Medicine and Supportive Care, UT MD Anderson Cancer Center, Houston, Texas.; ^7^ Memorial Sloan Kettering Cancer Center, New York, New York.; ^8^ African Palliative Care Association, Kampala, Uganda.; ^9^ Casa Sperantei, Transylvania University, Brasov, Romania.; ^10^ The George Washington University's Institute for Spirituality and Health (GWish), Washington, DC.; ^11^ WHO Collaborating Centre for Training and Policy on Access to Pain Relief, Pallium India, Trivandrum, Kerala, India.; ^12^ The Catholic University of Korea (CUK), Seoul, Korea.; ^13^ Icahn School of Medicine at Mount Sinai, New York, New York.; ^14^ Pontifical Academy for Life, Vatican City.

**Keywords:** advocacy, development, global, palliative care, position statement

## Abstract

***Background:*** The Pontifical Academy for Life (PAV) is an academic institution of the Holy See (Vatican), which aims to develop and promote Catholic teachings on questions of biomedical ethics. Palliative care (PC) experts from around the world professing different faiths were invited by the PAV to develop strategic recommendations for the global development of PC (“PAL-LIFE group”).

***Design:*** Thirteen experts in PC advocacy participated in an online Delphi process. In four iterative rounds, participants were asked to identify the most significant stakeholder groups and then propose for each, strategic recommendations to advance PC. Each round incorporated the feedback from previous rounds until consensus was achieved on the most important recommendations. In a last step, the *ad hoc* group was asked to rank the stakeholders' groups by order of importance on a 13-point scale and to propose suggestions for implementation. A cluster analysis provided a classification of the stakeholders in different levels of importance for PC development.

***Results:*** Thirteen stakeholder groups and 43 recommendations resulted from the first round, and, of those, 13 recommendations were chosen as the most important (1 for each stakeholder group). Five groups had higher scores. The recommendation chosen for these top 5 groups were as follows: (1) Policy makers: Ensure universal access to PC; (2) Academia: Offer mandatory PC courses to undergraduates; (3) Healthcare workers: PC professionals should receive adequate certification; (4) Hospitals and healthcare centers: Every healthcare center should ensure access to PC medicines; and (5) PC associations: National Associations should be effective advocates and work with their governments in the process of implementing international policy framework. A recommendation for each of the remaining eight groups is also presented.

***Discussion:*** This white paper represents a position statement of the PAV developed through a consensus process in regard to advocacy strategies for the advancement of PC in the world.

## Background

Every year, over 25.5 million people die with serious health-related suffering (SHS) associated with life-limiting and life-threatening conditions. An additional 35 million live with these conditions and SHS.^1^ Yet the vast majority of the world does not have access to adequate treatment and care and social support.

Palliative care (PC) helps relieve SHS by providing physical, psychosocial, and spiritual care to patients and their families. PC relieves “total pain” by shifting the often overly technical modern medical model to a holistic person-centered model of care.^2^

Estimates of unmet PC needs worldwide are around 26.8 million per year.^3^ Other data suggest an even greater need of up to 40 million people per year,^4^ with estimates reaching 61 million people around the globe suffering from SHS.^1^ Various additional studies have shown a deficit of PC demand to PC supply,^5–9^ highlighting a lack of access to PC as a major global health inequity issue.^10,11^

There has been a rising burden of noncommunicable diseases (NCDs) worldwide, and globally, NCDs cause 70% of all deaths^12^ and generate 93% of adult PC need, and nearly 80% of the global PC need is in low-to-middle income countries.^3,5^ Furthermore, the global population is aging, and this, partnered with the increased prevalence of NCDs and the persistence of other debilitating chronic and infectious diseases, reflects an alarming increase in need for PC provision at the global scale.^4^ In fact, studies estimate that by 2040, the proportion of people worldwide in need of PC will increase from 25% to 47%.^13^

This growing need is recognized by global health organizations; the World Health Organization (WHO) recently approved the 13th General Program of Work recognizing the “limited availability of [PC] services in much of the world and the great avoidable suffering for millions of patients and their families”^4,14^ and concluded with several recommendations for further PC development and support for global PC advocacy campaigns. Although research has shown that PC has steadily grown at the global level, the demand far outstrips supply, and this growth has been very uneven, with some countries having progressed very little over the past decade.^4–9^

The Catholic Church's appreciation for the PC as an approach to take care of the vulnerable is evident in its catechism, which includes the following statement “[Palliative care] represents a special form of disinterested charity, and as such, should be encouraged” (Catechism of the Catholic Church, n. 2279). Recently, Pope Francis shared with health professionals meaningful words on PC: “I encourage professionals and students to specialize in this type of assistance which is no less valuable for the fact that it is not life-saving. [PC] accomplishes something equally important: it values the person.”^15^

The Pontifical Academy for Life (PAV) is an academic institution of the Holy See (Vatican) dedicated to the promotion of human life, and, among other specific topics, the study issues in medical ethics. In 2017, the PAV launched an international project called “PAL-LIFE: An International Advisory Working Group on diffusion and development of palliative care in the world” to advise on how the Catholic Church could assist in continued PC development at the global level.^16^ This white paper represents a position statement of the PAV regarding PC, intended to be used for advocacy with local governments, healthcare organizations, leaders on the ground, and faith-based communities.

## Design

A process was developed to generate consensus among 13 PC experts on key recommendations for major stakeholders' groups, including ranking both the recommendations and the stakeholders' groups by importance, as well as providing suggestions for implementation.

The study was submitted and approved by the Clinical Research Ethics Committee of the University of Navarra.

### Selection of experts and definition of the process

The expert group was selected considering and balancing diverse geographical regions and professional backgrounds. Members included clinicians, ethicists, and health administrators working in academic centers or international and regional PC organizations and professing different faiths. The PAV initially chose three experts in PC advocacy with global expertise in PC development. In a second step, additional experts were added to the group through a snowball process of recommendations by at least 2 peer experts reaching a total of 13 persons considered to be experts in PC advocacy (“ad hoc group”). Two additional experts were invited in the last stage of the process based on the suggestion of several members of the group. Table 1 shows the members of the *ad hoc* group.

**Table 1.** Members of the PAL-LIFE Ad Hoc Group

*Name*	*Title/Institution*	*City*	*Country*
Alsirafy, Samy	Head of the Palliative Medicine Unit, Kasr Al-Ainy School of Medicine, Cairo University	Cairo	Egypt
Bruera, Eduardo	Chair, Department of Palliative Medicine and Supportive Care, UT MD Anderson Cancer Center	Houston	United States
Callaway, Mary V.	Board of Directors, IAHPC	Houston	United States
Centeno, Carlos	Director, ATLANTES Research Group, University of Navarra	Pamplona	Spain
De Lima, Liliana	Executive Director, International Association for Hospice and Palliative Care (IAHPC)	Houston	United States
Foley, Kathleen M.	Attending Neurologist Emeritus, Memorial Sloan Kettering Cancer Center	New York	United States
Luyirika, Emmanuel	Executive Director, African Palliative Care Association (APCA)	Kampala	Uganda
Mosoiu, Daniela	Director, Casa Sperantei, Assoc Prof. Transylvania University	Brasov	Romania
Pettus, Katherine	Advocacy Officer, IAHPC	Houston	United States
Puchalski, Christina	Director, The George Washington University's Institute for Spirituality and Health (GWish) Professor of Medicine GWU	Washington	United States
Rajagopal, M.R.	Director Pallium India, WHO Collaborating Centre for Training and Policy on Access to Pain Relief	Trivandrum	India
Sitte, Thomas	CEO Deutsche PalliativStiftung	Fulda	Germany
Yong, Jin-Sun	Director, The Catholic University of Korea (CUK), WHO Collaborating Centre for Training in Hospice and Palliative Care. Professor of Nursing, CUK	Seoul	South Korea

An initial face-to-face meeting was conducted at the venue of the PAV in Rome, in March 2017. The purpose of the meeting was to define the strategy and methodology for identification of the key recommendations to be determined by the *ad hoc* group. It was outlined as the project for a draft of a position statement (“white paper”) on PC advocacy containing recommendations for health policy planning and providing guidance to different stakeholder groups on how to advance the development of PC in countries and regions.

For the purposes of this project, the *ad hoc* group used the WHO definition of PC. The group also adopted the WHO public health strategy framework for PC.^17^

### Identification of stakeholder groups

In Round 1, experts of the *ad hoc* group were invited by e-mail to identify the most relevant stakeholder groups to which the recommendations would be directed to. These stakeholder groups were identified based on their key roles in their ability to promote PC development at national or regional levels in healthcare and/or society.

From the initial list, through a Delphi consensus process, members of the *ad hoc* group suggested new stakeholder groups or modified ones already in the list, resulting in a final list of 13 groups. Based on the field of expertise, each expert was assigned to a specific stakeholder group. Table 2 shows the stakeholder groups agreed upon by the *ad hoc* group.

**Table 2.** Ranking of Stakeholder Groups

*Stakeholder group*	*Points*	*Group K-mean*
Policymakers	122	103.4
Universities (academia)	111	
Healthcare workers	103	
Hospitals and healthcare centers	92	
Palliative care associations	89	
International organizations	71	52.4
Mass media	69	
Philanthropic organizations and charities	62	
Pharmaceutical authorities	59	
Patients and patient groups	53	
Spiritual care professionals	50	
Associations other than palliative care	29	
Pharmacists	26	

Scores on relative importance (range 1–156) and K-means for cluster analysis.

### Consensus process for the recommendations

In Round 2, each member was contacted by e-mail and requested to provide two to three recommendations for his/her corresponding stakeholder group. Each recommendation was accompanied by a statement, including rationale for the proposal, up to a maximum of 200 words. Recommendations were built upon the previous work and experience of the experts within their own or several other institutions to ensure best possible recommendations per stakeholder group.

In Round 3, all the recommendations were shared with the entire *ad hoc* group through an online survey tool (https://es.surveymonkey.com), and each member of the *ad hoc* group was asked to rank each recommendation on a Likert scale from 1 to 5 (1 being “not important at all” and 5 being “extremely important”). The average number of points for each recommendation was calculated.

The results were preliminarily presented in a PC conference organized by the PAV in Rome in March 2018 and, subsequently, discussed by the *ad hoc* group in a new face-to-face meeting with a subset of the experts. During this meeting, a thorough review of the recommendations and suggestions for implementation was conducted to improve wording.

In Round 4, 12 members of the *ad hoc* group reviewed their previous ratings and conducted another round of rankings of the stakeholder groups based on their perceived importance for PC development. Using a 13-point scale, points were assigned to each, according to the ranking given by each member [range: 12 (worst = 1 point per expert) to 156 (best = 13 points per expert)]. An exploratory K-means cluster analysis provided a classification of the stakeholders in different levels of importance for PC development. As final step in this Round 4, members of the *ad hoc* group were asked to provide suggestions for implementation for each of the recommendations.

### PAV endorsement of the recommendations and presentation of outcomes

The resulting recommendations from each stakeholder group were revised and agreed upon, then endorsed by the Board of Directors of the PAV. The endorsement will be announced during the plenary session of the annual meeting of the PAV (June 2018) as the official position of the Academy and as the recommendations of PAL-LIFE.

In this article we present the five highest ranked recommendations (“first-line”) with concrete suggestions for implementation. The additional eight recommendations for the remaining stakeholders' groups are presented as second-line interventions. All the recommendations are accompanied by a description, rationale, and bibliographic references. Additional recommendations discussed by the group, but not ranked within the highest scores for importance, are included in a report on the PAV website (www.academyforlife.va).

## Results

Thirteen stakeholder groups and 43 recommendations resulted from the first round, and of those, 13 recommendations were chosen as the most important (1 for each stakeholder group) and are presented in this study. These, plus the additional 30 recommendations are available in the PAV website.

Table 2 indicates the stakeholder groups and the total scores each received through the ranking (range 1–156). The K-means cluster analysis confirmed the existence of two levels in the ranking of the stakeholders' groups as follows: five groups had higher scores (closer to K-mean 103.4) and nine stakeholder groups lower ones (closer to K-mean 52.4).

First-line stakeholder groups for advocacy are (1) policy makers (in all units of government); (2) academia (universities and colleges); (3) healthcare workers; (4) hospitals and healthcare centers; and (5) PC professional associations. The recommendations for first-line stakeholders are included in Table 3 along with suggestions for their implementation. Second-line stakeholder groups (Table 4) are (6) international organizations; (7) mass media; (8) philanthropic organizations and charities; (9) pharmaceutical authorities; (10) patients and patient groups; (11) spiritual care professionals; (12) non-PC professional associations and societies; and (13) pharmacists. The recommendations can be seen in Table 4.

**Table 3.** Highest Ranked PAL-LIFE Group Recommendations to Stakeholders for the Promotion of Palliative Care and Suggestions for Implementation

*Position*	*Stakeholder and suggestions*
1.	**Policy makers:** Policy makers must recognize the societal and ethical value of palliative care and modify the existing healthcare structures, policies, and outcome measures to ensure universal access to palliative care for all patients in need. They must also take the steps to ensure an integrated health system, to allow a smooth flow of patients between the different levels of care, so that patients with complex problems may be referred to secondary and tertiary levels, as needed, and referred back to home care, if possible.
	Suggestions for implementation:
	• Involve national associations to advocate for palliative care
	• Advocate with local policymakers for access to palliative care as a human right
	• Link advocacy to other initiatives such as the movement of whole-person care, preventive medicine, and health promotion
	• Carryout a public awareness campaign focusing on needlessly suffering and the ethical responsibility of the government.
	• Include palliative care as a component of NCD national plans or strategies
2.	**Academia (universities and colleges):** All academic institutions offering degrees in healthcare-related fields should include mandatory palliative care courses as part of the undergraduate curricula.
	Suggestions for implementation:
	• Approve a national law where palliative care teaching is mandated
	• Develop standard curricula on team-based interdisciplinary palliative care
	• Palliative care curricula must combine theoretical and practical components integrated at the primary care level
	• Teach palliative care by clinically experienced faculties who have academic appointments
	• Funding for education programs should come from governments' healthcare educational budgets.
	• When palliative care is not taught, invite palliative care experts to deliver lectures on palliative care to create the demand
	• Adopt and implement the EAPC recommendations for the inclusion of palliative care in the undergraduate curricula for medical and nursing schools and implement the Initiation for System Transformation project (ITES) for countries throughout
	Latin America
	• Ensure training in the trainer courses, also in primary healthcare teaching.
3.	**Healthcare workers:** Healthcare professionals working in palliative care should receive appropriate certification while actively participating in continuing education to maintain the adequate competency levels
	Suggestions for implementation:
	• Reach out to the national boards of medicine and nursing and the Ministries of Health and education through National Associations to advocate for the recognition of palliative care as a specialty.
	• Establish a working group among members of the board of medicine and the board of nursing with palliative care experts in the country to determine the minimum level of competencies, knowledge and skills in palliative care, and years of dedication required to be recognized as palliative care professional.
	• Standardize health professional education with basic and specialty certification programs according to each country's process of healthcare professional official certification
4.	**Hospitals and healthcare centers:** Every hospital and healthcare center should ensure affordable access to palliative care medicines included in the WHO Model List of Essential Medicines, particularly to immediate-release oral morphine. It also should accept palliative care provision as a moral and ethical imperative.
	Suggestions for implementation:
	• Ensure training of all staff in the fundamentals of palliative care
	• Define a palliative care integration strategy for the hospital or Health Center
	• To establish a minimum dataset to monitor the quality of care in advance disease and end of life
5.	**Palliative care associations:** Representatives of national associations should be effective advocates and work with their governments in the process of implementing international policy framework, including Conventions, Resolutions, and Declarations in their countries (i.e., UNGASS outcome document, Agenda 2030, WHA Resolution).
	Suggestions for implementation:
	• Implement advocacy workshops with representatives of national associations to empower representatives of civil society so that they adopt the skills to do effective advocacy campaigns and strategies.
	• National associations have the power and legitimacy to request and demand from their governments the implementation of the international policies and frameworks which call for the inclusion of palliative care in the national policies and programs, the strengthening of NCD programs, and the adoption of the SDGs in the Agenda 2030.
	• Work to set national standards in palliative care, including primary and specialist palliative education, and training and work with both governmental and nongovernmental stakeholders to develop a national palliative care strategy integrated into universal healthcare.

NCDs, noncommunicable diseases; SDGs, sustainable development goals; WHO, World Health Organization.

**Table 4.** Other PAL-LIFE Group Recommendations to Stakeholder Groups for the Promotion of Palliative Care

*No.*	*Recommendations*
6	**To international organizations:** International organizations should encourage WHO Member States to develop policies and procedures to implement the WHA Resolution 67/19 as an integral part of their strategies and to implement the Agenda 2030 for Sustainable Development Goals, paying specific attention to the needs of children and older persons.
7	**To the mass media:** Mass media should be involved in creating a culture of understanding around advanced illness and the role of palliative care throughout the life course and as a component of UHC.
8	**To philanthropic organizations and charities:** Individuals and organizations involved in palliative care must engage, educate, and advocate for philanthropic organizations and charities to support palliative care development and implementation of services.
9	**To pharmaceutical authorities:** Morphine (preferably immediate release oral formulation) is the preferred medication for the treatment of moderate/severe cancer pain and palliative care and should be made available and accessible. No government should approve modified-release morphine, transdermal fentanyl patches, or slow release oxycodone without also guaranteeing widely available immediate-release oral morphine.
10	**To patients and patient groups:** Patients and patient groups could be of great help in developing and demanding a health literacy campaign for all patients with PC needs and their families to increase the knowledge and understanding of PC and its role in the decision-making process.
11	**To spiritual care professionals:** Religious institutions and spiritual care groups should work to include spiritual care—including ongoing assessment of spiritual distress and spiritual well-being—integrated into guidelines of care and as a component of routine palliative care provision.
12	**To professional associations and societies other than Palliative Care:** Nonpalliative care professional associations and societies should encourage human rights organizations to consider existing declarations and to implement strategies whose aim is advancing palliative care development worldwide within a human rights framework.
13	**To pharmacists:** Pharmacists should play an active role in palliative care teams by assessing the appropriateness of the medicines prescribed to patients, by ensuring timely dispensation, by educating the team members about pharmacological interactions, and by ensuring that patients and caregivers understand the prescribed regimen to ensure adherence to treatment.

PC, palliative care; UHC, Universal Health Coverage.

### Reflections for the advocacy of PC to first-line stakeholders' groups

#### Policy makers

Patients with chronic progressive diseases, such as cancer, congestive heart failure, chronic obstructive pulmonary disease, and HIV/AIDS, develop severe physical, psychosocial, and spiritual symptoms before death.^1,18^ There is strong evidence that PC is beneficial in reducing much of this suffering in patients, as well as psychosocial and spiritual or existential distress in families.^19^

There is strong evidence that these benefits are accompanied by a reduction in the total cost of care.^20^ Cost savings are achieved mainly by preventing unnecessary disease-oriented investigations and treatments, as well as hospitalizations in acute care hospitals and intensive care units.^21–25^ Value in healthcare results from the balance between benefits and costs. PC has demonstrated impact on both components of value.

#### Academia (universities and colleges)

According to the UN Committee on Economic, Social and Cultural Rights (CESCR), Member States are required to ensure universal access to PC. This obligation includes the duty to ensure that healthcare workers meet appropriate standards of education.^4^ Accordingly, the WHO urges Member States to integrate basic PC training into all undergraduate medical and nursing professional education.^11^ In other words, international law stipulates that governments and universities of Member States provide adequate training of healthcare workers pursuant to the principles laid out by the WHO.^26^

Studies also suggest that early and continuous student exposure to PC education is associated with positive attitudes and increased satisfaction toward PC among undergraduate medical students.^27^ Studies also demonstrate undergraduate nursing students' belief that PC training should be an essential component of their education, contributing favorably to both their personal and professional development.^4^

Complete integration of PC courses into all undergraduate curricula for future healthcare workers is both an obligation under international law and an evidence-based educational strategy.

#### Healthcare workers

In addition to requiring basic-level PC training for all undergraduate medical and nursing professional education, the WHO urges Member States to ensure intermediate-level training to all healthcare workers who routinely encounter patients with life-threatening illnesses and to fully integrate PC into healthcare in every setting, specifically highlighting community settings, and throughout the course of advanced illnesses. Member States are also required to provide specialist-level training to prepare healthcare professionals who will engage in more than routine PC practice.^4^ This means that healthcare workers must receive appropriate certification, acquiring competences that are required by the proper standards of certification. Specialist-level training is of particular importance in places where the role of PC specialists has not yet been institutionalized.

#### Hospitals and healthcare centers

Modern medical science, unfortunately, based increasingly on technology, has become so disease oriented as to neglect the human being. Health-related suffering is often ignored.

Persistent attempts at treating the disease, even in the face of futility of treatment, cause, in addition to physical, social, and mental suffering, financial difficulties and spiritual distress. In his address to participants in the Plenary of the PAV (Clementine Hall, March 5, 2015) Pope Francis said, “I therefore welcome your scientific and cultural efforts to ensure that PC can reach all those who need it. I encourage professionals and students to specialize in this type of assistance, which has no less value on account of fact that it does not save lives. PC recognizes something equally important, the value of person.”^15^

The World Health Assembly in its landmark Resolution of 2014^4^ called upon all Member States to integrate PC in Healthcare at all levels (primary, secondary, and tertiary) across the continuum of care (from the time health-related suffering starts until the death of the patient and continuing thereafter in the form of bereavement support for the family).

#### PC associations

Patients who require PC often have diverse and overlapping illnesses and may be staying at home, in long-term care facilities, nursing homes, and hospitals. Delivery of holistic services to patients requires multidisciplinary teams which may work in the national/public health system, the church, or nongovernment sectors.^4^

These teams need to plan their interventions based on the needs of the patient, whether adult or child, and the patient's family.^28^ To develop the skills and improve their knowledge, the members of the multidisciplinary teams rely on guidelines and recommendations from PC associations and societies that often work with governments, other civil society agencies, donors, and promoters of PC to set up functional capacity building, service delivery, and research networks.^29^ These build a system that can reach even the most disadvantaged communities not reached by conventional healthcare systems.^30^

### Reflections for the advocacy of PC to second-line stakeholders' groups

#### International organizations

Recognizing that more than 75% of the world has no access to PC services, WHO Member States unanimously adopted WHA Resolution 67/19 in 2014. In 2015, UN Member States unanimously adopted Agenda 2030 for Sustainable Development in 2015 with the pledge to “leave no one behind.” Leaving no one behind means that UN Member States and agencies must collaborate to develop integrated, human rights based policies and procedures to realize their key public health outcomes. Human rights based public health policies make integrated person-centered services available to all citizens, migrants, and refugees of all ages in all settings: home, hostel or hospice, rural or urban clinic, hospital, and long-term settings such as nursing homes and prisons.^3,16,31–38^

#### Mass media

There is a misconception about PC both among the general public and among healthcare professionals that PC is synonymous with end-of-life care.^39^ PC is not just for the dying. With this understanding comes an imperative for patients to receive PC earlier in their disease trajectory.^40^ This requires a cultural shift that starts with physicians to the general population.

#### Philanthropic organizations and charities

PC must be integrated into national health systems around the world. National governments have not provided adequate financing to support PC development, and nongovernmental organizations, professional organizations, foundations, faith-based organizations, charities, charitable trusts, and development agencies have played important roles in the development of hospice and PC at the international and community levels, providing both medical and social support.

With the potential for governments to provide universal health coverage (UHC) and a basic package for PC, all donor organizations must work with PC providers to develop innovative educational and social support systems.^41–44^

#### Pharmaceutical authorities

Morphine is recommended by the WHO as the first-line strong opioid for the management of moderate-to-severe cancer pain in adults and children.^45–48^ Although it is available in different formulations,^49^ it is recommended that the availability of cheap immediate-release oral morphine is a priority due to reasons such as affordability and flexibility in use.^50^ Although other, newer strong opioids should also be made available, availability of these newer opioids should not be considered as a replacement to availability of morphine.

#### Patients and patient groups

Health illiteracy, even in countries where PC is well developed, is an obstacle for early integration of PC, which improves therapy.^40^ Mistakenly, some patients may perceive that alleviating symptoms is a way to hasten death.^51^ There is significant health illiteracy, and patients and families are not aware that PC can be given concurrently with active disease oriented therapies. Education targeted to these groups can help to dispel the myths about PC as hastening death or only a care approach for dying patients.

#### Spiritual care professionals

The WHO has recognized spiritual care as a required element of PC. Spiritual distress (spiritual or existential suffering) needs to be addressed by all members of the team to provide the best quality care for patients and families and to help relieve suffering of patients and families. Several U.S. and international consensus conferences have developed definitions and models for addressing spiritual distress in the clinical setting.^52^

Religious leaders should advocate for the inclusion of interprofessional spiritual care in PC and advocate for appropriate training of all clinicians in providing spiritual care to patients and families, as well as developing, training, and helping to sustain adequate staffing of healthcare chaplains in all health settings.^53,54^

#### Non-PC professional associations and societies

Acknowledgment of pain relief and PC as a human right have been widely declared by many institutions and organizations.^4,10,49,55,56^

#### Pharmacists

PC patients often need to take multiple medications simultaneously and, as a result, have an increased risk of drug interactions and drug-related problems of essential medicines for PC. Pharmacists have more knowledge of medications and their effects than any other member of the healthcare team and are, therefore, the best equipped to detect possible problems and make the appropriate recommendations.^57,58^

## Discussion

This article presents the consensus of 13 PC experts from around the world, in line with the PAL-LIFE objectives, on what are considered the most important recommendations to 13 groups of stakeholders to help advance PC development. Some of the recommendations are applicable to several stakeholder groups (i.e., recommendation for pharmaceutical authorities on morphine availability should also be directed to lawmakers, administrators, pharmaceutical manufacturers, dealers, and PC advocates or recommendation to universities should also be presented to healthcare workers and educators).

Many of the items presented in this study require a coordinated approach. Globally, a majority of patients die with severe pain without having ever received a single dose of morphine or other opioid analgesic. To address this tragic situation, it is important to harmonize the need for increased access to opioids for pain treatment, while taking into consideration the abuse potential and adverse effects. This requires a coordinated approach among policymakers, universities, pharmacists, and professional associations so that safety measures are put in place for the goals to be achieved.

The recommendations in this White Paper focus on crucial issues. However, optimal situations may require more comprehensive and broader recommendations (i.e., the recommendation for pharmaceutical authorities on morphine availability should be accompanied by a statement clarifying that more than one low priced opioid is needed since up to 80% of patients may need opioid rotation at some point, even though only morphine is specifically recommended in this study). The critical issue is that governments should take the necessary steps to ensure access to PC medicines included on the WHO Model List of Essential Medicines, including morphine as the gold standard and all the others in the List. Similarly, some recommendations may not capture the importance of spiritual care that is equally important to the physical and psychosocial domains. Spiritual, religious, and existential concerns are also dimensions which require care and should be addressed, registered, monitored, and managed by the PC team.

One limitation of this article is that it is based on the consensus of a small (13) group of PC experts and later approved by the board of directors of the PAV. A larger group could have probably resulted in additional stakeholder groups which would have broadened the scope of this position article. For these reasons, the group strongly recommends considering the recommendations broadly, while taking into account the additional 30 agreed-upon recommendations available on the website of the PAV (www.academyforlife.va).

This white paper represents a position statement of the PAV with regard to PC. Caring for the sick has been part of the missionary activity of the Catholic Church since its inception. The Church refers to PC as “a special form of disinterested charity. As such it should be encouraged” (Catechism of the Catholic Church, n. 2279). The Magisterium of the Catholic Church has intervened several times in recent years to emphasize the dignity and preciousness of each human being, even of those who are afflicted with serious or terminal illnesses. Recently, Pope Francis described PC as “an expression of the truly human attitude of taking care of one another, especially of those who suffer. It is a testimony that the human person is always precious, even if marked by illness and old age. […] Thus, I appreciate your scientific and cultural commitment to ensuring that palliative care may reach all those who need it. I encourage professionals and students to specialize in this type of care that is no less valuable for the fact that it ‘is not life-saving.’ PC accomplishes something equally important: it values the person.”^15^

The Christian movement, consistent with the teachings of Jesus of caring for the destitute, the vulnerable, and the poor, has developed and built large care networks which include hospitals, clinics, and health centers throughout the world. Faith-based hospitals and healthcare institutions, from local clinics to tertiary research institutions, are all sites where PC fits in as part of the concept of care and solidarity, as well as a component of care within the health system. In many countries, regardless of the most prevalent professed faith, a significant number of healthcare facilities are operated by the Catholic Church and other Christian denominations. With such a large network, the Church has the opportunity to lead a major movement to relieve the suffering of millions of patients and their families.

This White Paper may be used as a checklist for countries or regions to identify and implement basic strategies to improve the care for patients and families with PC needs. It can also serve as the basis for development of a more comprehensive list of recommendations adapted to the institutions or groups within each stakeholder group or specific geographical contexts. It will be undoubtedly useful for advocacy with local governments, faith-based communities, and others.

In summary, this White Paper emphasizes the responsibility of healthcare systems and stakeholders to recognize access to pain relief and PC as a basic right of the person and the family and the responsibility of all elements of the healthcare system. For this, it is necessary to recognize health as not only an absence of disease but also as physical, emotional, social, and spiritual well-being, which can be optimized only by making essential PC medicines available, governments integrating PC into their healthcare plans and UHC, and developing public and professional education, as well as clear frameworks for implementing this care to prevent needless suffering. The support of faith-based and philanthropic organizations, nongovernmental and governmental actors, and human rights organizations is needed to support PC integration. In short, a civil society response is needed.
